# Health status and lifestyle factors as predictors of depression in middle-aged and elderly Japanese adults: a seven-year follow-up of the Komo-Ise cohort study

**DOI:** 10.1186/1471-244X-11-20

**Published:** 2011-02-07

**Authors:** Hisashi Tanaka, Yosiaki Sasazawa, Shosuke Suzuki, Minato Nakazawa, Hiroshi Koyama

**Affiliations:** 1 Department of Public Health, Gunma University Graduate School of Medicine, Maebashi, Japan; 2 Faculty of Education, University of the Ryukyus, Okinawa, Japan; 3 NPO International Ecohealth Institute, Isesaki, Japan

## Abstract

**Background:**

Depression is a common mental disorder. Several studies suggest that lifestyle and health status are associated with depression. However, only a few large-scale longitudinal studies have been conducted on this topic.

**Methods:**

The subjects were middle-aged and elderly Japanese adults between the ages of 40 and 69 years. A total of 9,650 respondents completed questionnaires for the baseline survey and participated in the second wave of the survey, which was conducted 7 years later. We excluded those who complained of depressive symptoms in the baseline survey and analyzed data for the remaining 9,201 individuals. In the second-wave survey, the DSM-12D was used to determine depression. We examined the risks associated with health status and lifestyle factors in the baseline survey using a logistic regression model.

**Results:**

An age-adjusted analysis showed an increased risk of depression in those who had poor perceived health and chronic diseases in both sexes. In men, those who were physically inactive also had an increased risk of depression. In women, the analysis also showed an increased risk of depression those with a BMI of 25 or more, in those sleeping 9 hours a day or more and who were current smokers. A multivariate analysis showed that increased risks of depression still existed in men who had chronic diseases and who were physically inactive, and in women who had poor perceived health and who had a BMI of 25 or more.

**Conclusions:**

These results suggest that lifestyle and health status are risk factors for depression. Having a chronic disease and physical inactivity were distinctive risk factors for depression in men. On the other hand, poor perceived health and a BMI of 25 or more were distinctive risk factors for depression in women. Preventive measures for depression must therefore take gender into account.

## Background

Depression is a common mental disorder that causes psychological anguish and has a substantial impact on one's private and public life [1]. Mental health has been incorporated into the international health policy agenda as a top priority and depression is included in the three leading causes of burden of disease in 2030 estimated by World Health Organization (WHO) [2]. To help prevent depression, a variety of studies on the risk factors for depression have been conducted worldwide [3,4]. Several studies have found that a wide variety of factors, such as socio-demographics, health status, lifestyle and social networks, are involved in the incidence of depression. Studies of non-clinical depression have investigated a variety of risk factors for depression using the Center for Epidemiologic Studies Depression Scale (CES-D) [5]. They have reported that, in females, not having a spouse, living alone, having a disability, having insufficient social support, developing a new health condition, perceiving one's health as poor and having a limited ability to perform physical activities significantly increase the risk of depression [6-8]. Other studies using a diagnostic evaluation based on the diagnostic criteria of the Diagnostic and Statistical Manual of Mental Disorders, 4^th ^Edition (DSM-IV) have reported that insomnia, hypersomnia, other sleep complaints, female gender, social isolation, poor self-perceived health and impairment of functional abilities increase the risk of depression [9,10].

As in other industrialized countries, depression has become the most common mental disorder in Japan. Numerous Japanese studies have examined depression in the elderly because Japan is a leader in longevity and possesses an aging society [11]. It has been suggested that lifestyle and health status associate with depression. However, for middle-aged adults, the group with the highest suicide rate in Japan [12], there are a limited number of cross-sectional studies on the risk factors related to depression [13,14]. Miyaji et al. [13] using the CES-D for community residents reported that individuals with good self-perceived health who got more than six hours of sleep per night tended to have a low risk of depression. A study of workers [14] using Zung's Self-Rating Depression Scale [15] reported significantly lower depression scores in males who ate breakfast regularly, engaged in regular physical activity and consumed moderate quantities of alcohol, as well as in non-smoking females who slept 7 to 8 hours per night regularly and engaged in regular physical activity.

To prevent depression, it is necessary to clarify the nature of association between the risk factors and future development of depression. Considering the results of previous studies, we chose three health status items of perceived health status, chronic diseases and body mass index (BMI) and four lifestyle factors including hours of sleep per night, smoking, alcohol consumption and physical activity. In the present study we investigated these factors in non-depressive subjects and analyzed the association with future development of depression in a large-scale longitudinal setting. To understand the underlying factors of developing depression is possibly the first step to prevent depression.

## Methods

### Study cohort

**Figure 1:**
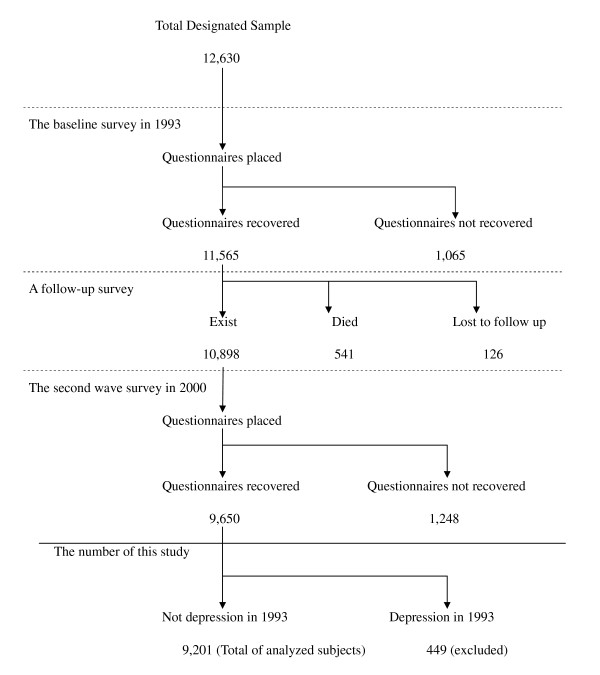
**Number of samples of Komo-Ise cohort 1993-2000**.

The Komo-Ise study [16,17] included 12,630 middle-aged and elderly persons. The original goal of the study was to examine the relationship between lifestyle and sociodemographic risk factors and mortality. Figure 1 shows the number of individuals in the Komo-Ise cohort from 1993-2000. Subjects in the Komo-Ise study were men and women aged 40-69 years living in the village of Komochi and the downtown area of the city of Isesaki who were identified based on the municipal resident registration file in 1993.

Baseline survey: In Komochi, a residents' association in the village distributed self-report questionnaires to households where the potential respondents resided in January 1993. In Isesaki, the same questionnaires were distributed in October 1993 via a health promotion committee. The questionnaire was left at the household to be completed, sealed and collected. There were 4,501 respondents in Komochi (response rate: 92.3%) and 7,064 in the downtown area of Isesaki (response rate: 91.1%). Therefore, responses were obtained from a total of 11,565 individuals: 5,630 men (response rate: 91%) and 5,935 women (response rate: 91%).

Registration follow-up: The subjects were followed from January 1993 to October 2000. Information on deaths and changes of residence was obtained from data in the municipal resident registration files in each locality. During the follow-up period, 541 deaths (4.7%) were confirmed to have occurred. Subjects who failed to respond by mail or who were not eligible to respond were defined as lost to follow-up (n = 126, 1.1%).

Second-wave survey: The municipal staffs of Komochi and Isesaki distributed the second wave of the questionnaire in November 2000. The questionnaire was mailed to individuals who had moved away. Responses were obtained from 9,650 of the 10,898 subjects (88.5%).

The Komo-Ise study was approved by the Epidemiologic Research Ethics Committee of Gunma University Faculty of Medicine, Maebashi, Japan.

## Methods

Questionnaires: The baseline questionnaire elicited information on respondents' demographic characteristics, health status, lifestyle factors and social networks, and also included the Todai Health Index (THI) [18], which quantitatively represents mental and physical complaints. The Japanese-language version of a 1999 survey questionnaire used as part of the Alameda County Study [19] was used for the second-wave survey in 2000. Questions in English were translated by bilingual native speakers according to the process of translation and back-translation. The questionnaire consisted of items on socio-demographics, health (chronic diseases, daily activities, etc.), lifestyle, social networks, mental health, abuse and socioeconomic status.

### Depression

The 12-item scale for depression from the Diagnostic and Statistical Manual of Mental Disorders (DSM-12D) [20] was used to detect depression in the second-wave survey. This is a self-administered questionnaire that mirrors the diagnostic criteria for a major depressive episode in the DSM-IV. The probe statement inquires as to whether the respondent has experienced a particular symptom of depression nearly every day for the past two weeks. Subjects reporting five or more symptoms of depression, including depressed mood or anhedonia during their usual activities, are diagnosed with a major depressive episode. This method for detecting depression was also used in the Alameda County Study [19,21-23].

### Covariates

Based on the items in the 1993 baseline survey, following three health status items and four lifestyle items were used as covariates.

### Health status items

Three items addressed health: perceived health status, chronic diseases and BMI (<18.5, 18.5-25, >25). Perceived health status was assessed by asking, "What is your current health condition: excellent, good, fair, poor, or very poor?" The answers were coded as excellent/good/fair versus poor/very poor.

### Lifestyle items

Four items addressed lifestyle factors: hours of sleep per night (<6 hours, 6-9 hours, >9 hours) [24], smoking, alcohol consumption and physical activity. Alcohol consumption was assessed by asking, "Do you drink a lot of alcoholic beverages?" with possible answers of "yes," "only a little," or "never drink."

### Adjusted items

The adjusted socio-demographic items were as follows: age (grouped in five-year intervals), area (Komochi/downtown Isesaki), education (junior college, college, higher/other), occupation (unemployed, salaried employee, self-employed, agriculture and forestry), and social network items. The social network was evaluated through information on the following: 1) marital status, 2) household size, 3) enjoyment of good fellowship with neighbours, 4) participation in activities, and 5) having close friends. The respective questions were as follows: 1) What is your current marital status? (Married/single, with divorced and widowed coded as single); 2) How many people do you live with? (Number; dichotomized for analysis into "living alone" versus "two or more persons in the household"); 3) Do you enjoy good fellowship with your neighbours? (Yes/no); 4) How often do you take part in hobbies, club activities, or community groups? (Very often/often/sometimes/never); and 5) When you are in need, do you have close friends you can turn to? (Yes/no).

### Subjects of the current analysis

The 9,650 respondents to the second-wave survey in 2000 were established as the investigation subjects. We excluded 449 respondents: those with a THI score for depression (THI-D) of 22 points or higher in a possible range of 10-30, indicating a high level of depressive symptoms [25] (373 subjects, 176 men and 197 women), and those who reported having a mental illness as a chronic disease (76 subjects, 21 men and 55 women). This left 9,201 subjects in the final sample for analysis (4,326 men, 4,875 women).

### Statistical analysis

Using two logistic regression models adjusted for age alone (model 1) and for age, area, education, occupation, social network (marriage, household, neighborhood, participation, and friends) (model 2), risk factors for major depression in 2000 were evaluated in terms of the odds ratio (OR) and its 95% confidence interval (CI). SPSS (Version 11.5J) was used for statistical analysis.

## Results

Table 1 shows the characteristics and the social network variables for the subjects included in the analysis and the number cases of depression in 2000 by sex. For men, the prevalence of depression was significantly different between those who were married (1.6%) and those who were unmarried (3.1%) and between those living with other people (1.7%) and those living alone (4.4%). For women, the prevalence of depression was significantly different between those who reported having friends (1.5%) and those who reported having no friends (2.5%).

**Table 1:** The number of analysis subject's characteristics and the number of depression in 2000.

	Men				Women			
		
	N	%	Depression(%)	*p*-value	N	%	Depression(%)	*p*-value
Total			63(1.7)				79(1.9)	
								
Age class				*p *= 0.76				*p *= 0.17
40-44 years	783	18.1	15(2.1)		752	15.4	14(2.0)	
45-49 years	707	16.3	9(1.4)		791	16.2	12(1.7)	
50-54 years	752	17.4	14(2.1)		796	16.3	12(1.7)	
55-59 years	693	16.0	11(1.8)		929	19.1	14(1.8)	
60-64 years	838	19.4	9(1.4)		926	19.0	9(1.3)	
65-69 years	553	12.8	5(1.2)		681	14.0	16(3.4)	
								
Area				*p *= 0.32				*p *= 0.35
Rural	1,872	43.3	22(1.5)		1,923	39.4	33(2.1)	
Urban	2,454	56.7	41(1.9)		2,952	60.6	44(1.7)	
								
Education				*p *= 0.37				*p *= 0.26
Less than high school and vocational or special school								
	3,532	84.6	49(1.7)		4,347	93.4	66(1.8)	
Junior college and college or higher								
	643	15.4	13(2.2)		307	6.6	8(2.7)	
								
Occupation				*p *= 0.13				*p *= 0.52
Any kind of occupation	4,048	96.7	56(1.6)		3,210	72.3	49(1.8)	
No occupation	138	3.3	4(3.5)		1,229	27.7	22(2.1)	
								
Marriage				*p *< 0.05				*p *= 0.62
Married	3,661	89.3	51(1.6)		3,780	82.2	56(1.7)	
Unmarried	440	10.7	11(3.1)		820	17.8	16(2.4)	
								
Household				*p *< 0.05				*p *= 0.85
More than 2	4,176	97.4	59(1.7)		4,592	95.2	73(1.9)	
Living alone	111	2.6	4(4.4)		231	4.8	3(1.7)	
								
Neighborhood				*p *= 0.65				*p *= 0.39
Yes	1,590	37.7	24(1.8)		2,328	49.0	40(2.1)	
No	2,626	62.3	37(1.6)		2,424	51.0	36(1.7)	
								
Participation				*p *= 0.42				*p *= 0.39
Yes	3,165	75.3	44(1.6)		3,501	73.8	52(1.7)	
No	1,036	24.7	18(2.0)		1,246	26.2	23(2.2)	
								
Friends				*p *= 0.45				*p *< 0.05
Yes	2,526	60.3	33(1.5)		3,346	70.9	44(1.5)	
No	1,661	39.7	27(1.9)		1,375	29.1	29(2.5)	

*Subjects with missing values were excluded from each calculation of proportion.

Depression (%): Number of depression in 2000 (%)

Table 2 shows the health status variables for the subjects in the analysis and the number of cases of depression in 2000. For men, the prevalence of depression was significantly different between those responding "excellent", "good" or "fair" (1.5%) and those responding "poor" or "very poor" (4.6%) to the perceived health status variable and between those without (1.1%) and those with (2.6%) chronic disease. For women, the prevalence of depression differed significantly according to all of the variables. The prevalence of depression was 1.7 for those with excellent, good or fair perceived health status versus 5.3 for those with poor and very poor perceived health status. In addition, the prevalence of depression was 1.3 for those with no chronic disease and 3.0 for those with chronic disease. Furthermore, the prevalence of depression was 1.6 for those with a BMI in the 18.5-25 range, 2.1 for those with a BMI < 18.5 and 2.9 for those with a BMI of >25.

**Table 2:** The number of analysis subject's health status items, and the number of depression in 2000.

	Men				Women			
		
	N	%	Depression(%)	*p*-value	N	%	Depression(%)	*p*-value
**Health status**								
								
Perceived health status				*p *< 0.001				*p *< 0.001
Excellent, good, fair	4,036	93.9	53(1.5)		4,568	94.2	64(1.7)	
Poor, very poor	264	6.1	10(4.6)		279	5.8	13(5.3)	
								
Chronic disease				*p *< 0.01				*p *< 0.001
No	2,843	67.4	27(1.1)		3,119	65.9	35(1.3)	
Yes	1,373	32.6	30(2.6)		1,617	34.1	41(3.0)	
								
Body mass index				*p *= 0.79				*p *< 0.05
18.5-25	3,192	74.6	46(1.7)		3,474	72.3	46(1.6)	
<18.5	147	3.4	3(2.5)		228	4.7	4(2.1)	
25≦	942	22.0	13(1.6)		1,103	23.0	27(2.9)	

*Subjects with missing values were excluded from each calculation of proportion.

Depression (%): Number of depression in 2000 (%)

Table 3 shows the lifestyle variables for the subjects in the analysis and the number of associated cases of depression in 2000. For men, the prevalence of depression was significantly different between those who reported no (2.5%), light (1.2%) and heavy (2.1%) alcohol consumption and between those who often and sometimes (1.0%) and those who never (2.3%) engaged in physical activity. For women, the prevalence of depression was significantly different between those who slept 6-9 hours (1.8%), <6 hours (3.0%) and 9 hours < (6.4%). The other lifestyle variables did not associate significantly with the prevalence of depression.

**Table 3:** The number of analysis subject's lifestyle items, and the number of depression in 2000.

	Men				Women			
		
	N	%	Depression(%)	*p*-value	N	%	Depression(%)	*p*-value
**Lifestyle**								
								
Hours of sleep				*p *= 0.43				*p *< 0.05
6-9 hours	3,965	93.9	56(1.7)		4,431	93.0	66(1.8)	
<6 hours	119	2.8	3(2.9)		276	5.8	7(3.0)	
9 hours <	140	3.3	3(2.9)		59	1.2	3(6.4)	
								
Smoking				*p *= 0.90				*p *= 0.07
Never	1,133	29.3	17(1.8)		4,121	88.9	58(1.6)	
Past	732	18.9	10(1.6)		85	1.8	2(2.9)	
Current	2,004	51.8	32(1.9)		427	9.2	12(3.3)	
								
Alcohol consumption				*p *< 0.05				*p *= 0.62
Never	872	20.6	18(2.5)		2,739	57.5	48(2.1)	
Light	2,260	53.5	23(1.2)		1,915	40.2	27(1.6)	
Heavy	1,095	25.9	20(2.1)		110	2.3	2(2.0)	
								
Physical activity				*p *< 0.01				*p *= 0.07
Often, sometimes	1,995	47.3	17(1.0)		1,964	41.2	23(1.4)	
Never	2,224	52.7	44(2.3)		2,801	58.8	52(2.2)	

*Subjects with missing values were excluded from each calculation of proportion.

Depression (%): Number of depression in 2000 (%)

Table 4 shows related risk factors by sex according to both models. For men, Model 1 indicated that poor perceived health and suffering from chronic diseases were significant risk factors for the development of depression. The ORs and 95% CIs for the poor perceived health and chronic disease variables were 2.66, 1.54-4.57, and 3.09, 1.54-6.18, respectively. Moreover, model 2 indicated that having chronic diseases was a significant risk factor, OR: 2.19 and 95% CI: 1.16-4.14. Both model 1 (OR: 2.39 and 95% CI: 1.36-4.21) and model 2 (OR: 2.58 and 95% CI: 1.31-5.05) indicated that a lack of physical activity was a significant risk factor for development of depression after 7 years; no such risk factors were found to be associated with the other lifestyle variables.

**Table 4:** Odds ratios of depression in 2000 for variables in 1993.

	Men				Women			
Variable	Model 1		Model 2		Model 1		Model 2	
	OR	(95% CI)	OR	(95% CI)	OR	(95% CI)	OR	(95% CI)
**Health status**								
Perceived health status								
Excellent, good, fair	1.00		1.00		1.00		1.00	
Poor, very poor	3.09^b^	(1.54-6.18)	2.02	(0.88-4.65)	3.32^b^	(1.80-6.14)	2.39^a^	(1.09-5.24)
Chronic disease								
No	1.00		1.00		1.00		1.00	
Yes	2.66^b^	(1.54-4.57)	2.19^a^	(1.16-4.14)	2.38^b^	(1.48-3.82)	1.52	(0.86-2.70)
Body mass index								
18.5-25	1.00		1.00		1.00		1.00	
<18.5	1.60	(0.49-5.26)	1.40	(0.39-4.94)	1.31	(0.46-3.68)	1.23	(0.37-4.13)
25≦	0.92	(0.50-1.72)	0.62	(0.28-1.36)	1.89^b^	(1.17-3.08)	1.90^a^	(1.08-3.33)
								
**Lifestyle**								
Hours of sleep								
6-9 hours	1.00		1.00		1.00		1.00	
<6 hours	1.72	(0.53-5.58)	1.12	(0.25-4.95)	1.66	(0.75-3.66)	1.48	(0.57-3.88)
9 hours <	2.00	(0.61-6.63)	2.02	(0.43-9.50)	3.78^a^	(1.13-12.70)	1.13	(0.14-8.98)
Smoking								
Never	1.00		1.00		1.00		1.00	
Past	0.88	(0.40-1.94)	0.84	(0.34-2.09)	1.67	(0.40-6.99)	2.65	(0.61-11.59)
Current	1.01	(0.55-1.83)	1.01	(0.50-2.05)	2.04^a^	(1.08-3.85)	2.09	(0.97-4.51)
Alcohol consumption								
Never	1.00		1.00		1.00		1.00	
Light	0.46	(0.25-0.86)	0.54	(0.26-1.13)	0.79	(0.49-1.28)	0.67	(0.37-1.19)
Heavy	0.81	(0.42-1.55)	0.99	(0.46-2.11)	1.01	(0.24-4.24)	0.39	(0.05-3.08)
Physical activity								
Often, sometimes	1.00		1.00		1.00		1.00	
Never	2.39^b^	(1.36-4.21)	2.58^b^	(1.31-5.05)	1.59	(0.96-2.61)	1.23	(0.69-2.21)

Model 1 adjusted for age (5-year age categories). Model 2 adjusted for age (5-year age categories), area (rural/urban), education (compulsory education, high school and vocational or special school/junior college and college or higher), occupation (any kind of occupation/no occupation), social network (marriage; married/unmarried, household; more than 2/living alone, neighborhood; yes/no, participation; yes/no, friends; yes/no).

OR, odds ratio; 95% CI, 95% confidence interval. ^a^...P < 0.05, ^b^...p < 0.01.

For women, Model 1 indicated that poor perceived health and suffering from chronic diseases were significant risk factors for the development of depression. The ORs and 95% CIs for the poor perceived health and the chronic diseases variables were 3.32, 1.80-6.14, and 2.38, 1.48-3.82, respectively. Moreover, Model 2 indicated that having poor perceived health was a significant risk, OR: 2.19 and 95% CI: 1.16-4.14. Both model 1 (OR: 1.89 and 95% CI: 1.17-3.08) and model 2 (OR: 1.90 and 95% CI: 1.08-3.33) indicated that a BMI of >25 was a significant risk factor for development of depression. Model 1 also indicated a significant increased risk in women who slept more than 9 hours per night (OR: 3.78 and 95% CI: 1.13-12.70) and in women who were current smokers (OR: 2.04 and 95% CI: 1.08-3.85). In Model 2, no lifestyle variables were associated with a significantly increased risk for the development of depression although the odds ratio of smoking was almost same value as in Model 1.

## Discussion

### Health status

In this study, we showed that health status was a significant risk factor for the development of depression in both men and women. Having chronic diseases was a significant risk factor for depression in men, whereas poor perceived health was a significant risk factor in women. It has been reported that women with depression have a greater variety of depressive symptoms [26,27], regardless of the presence of chronic diseases.

The results showed gender difference of association of BMI with the development of depression. A previous large-scale study reported that major depressive disorder was associated with a high BMI in women and a low BMI in men [28]. The "jolly fat" hypothesis [29] was substantiated only in men by another study [30], in which the authors speculated that the "jolly fat" hypothesis may not apply to women because they are more likely than men to be stigmatized for being overweight or obese in industrialized societies. Results of meta-analysis of community-based studies [31] and longitudinal studies [32] also show the gender difference. The present study is a longitudinal investigation over 7 years and shows that a BMI of 25 or more in women is a critical factor in the future development of depression. Considering the result of the association between obesity and depression is important to prevent and treat depression of obese women and also prevent and treat obesity in women.

### Lifestyle

The result of Model 1 showed that sleeping longer than 9 hours per night was a risk factor for the development of depression in women. However, Model 2 failed to show such association. The discrepancy of the results implies that the adjusted variables used in model 2 were, al least partly, possible conflicting factors. Previous studies have reported that hypersomnia due to a diagnosed sleep disorder is a risk factor for the development of depression [9,33,34]. The number of hours spent sleeping is expected to differ depending on the individual and the culture and practices of the population to which he or she belongs, so the current results do not necessarily indicate that sleeping for more than 9 hours per night is a risk factor for the development of depression in women. In addition, a greater number of hours spent sleeping may raise the possibility of low sleep efficiency. However, an improved understanding of what specific sleep duration is a risk factor for the development of depression may make the prevention of depression more effective.

This study found that smoking is a risk factor for the development of depression in women. The result of Model 2 was not significant but showed that smoking is a weak risk factor for the development of depression. The association between smoking and depression has been previously reported [35-39]. In a survey of a Mexican population, Benjet et al. [40] found that the depression scores of male smokers were not significantly higher than those of male non-smokers, but the depression scores of female smokers were higher than those of female non-smokers. They hypothesized that sex-related differences in the social acceptance of smoking, as well as in nicotine metabolism, might influence the risk of depression and suggested that smoking is less socially acceptable for women than for men in Mexico. In Japan, Mino et al. [41] reported that smoking has a greater effect on mental health in women than in men. Similarly, the smoking rate among the current subjects was significantly higher for men (men, 53.2%; women, 10.2%; p < 0.01), suggesting that smoking was not as socially acceptable for women as for men in Japan. Stigmatisation of smoking women could lead to low self-esteem and the development of depression. It is needed to understand such underlying factors related to the association between smoking and development of depression.

In this study, alcohol consumption was not a risk factor for the development of depression in men or women. Several studies have consistently indicated a strong association between alcohol dependence or alcoholism and depression, and alcohol dependence or alcoholism is frequently co-morbid with depression [42-44]. However, these studies were not clear as to whether a drinking habit is a risk factor for the development of depression in the general population. Haynes et al. examined whether excessive alcohol consumption was a risk factor for depression in the general population, but found it not to be associated with the onset of depression [45]. Our results also suggest that drinking habits in non-depressive population are not risk factors for the future development of depression.

Several previous longitudinal studies have shown that moderate physical activity has a beneficial effect on depression, regardless of gender [19,46,47]. A clinical study has also demonstrated the anti-depressive effects of physical activity in both men and women [48]. However, in the present study, a lack of physical activity was a risk factor for the development of depression in men but not in women. Using the General Health Questionnaire (GHQ), Ohta et al. [49] also found that the GHQ score decreased with increasing levels of leisure-time exercise and with commuting to work by either walking or cycling in men but not in women. These studies suggest that leisure-time exercise and physical activity while commuting to work are associated with better mental health in men. The result of meta-analysis suggests that even low doses of physical activity may be protective against depression [50].

### Limitations

The first limitation of this study is that we used a self-report questionnaire to obtain information about the health and lifestyle factors. So we observed perceived recognitions of the subjects to the question items. This implies that affective mode possibly influenced the answers to the items as a confounding factor. The second limitation is a non-response bias due to the likely lack of responses to the second-wave survey from those with severe depressive symptoms. This bias might have led to a possible decrease of depression incidence and an inappropriate estimate of the risk posed by the various factors. The third limitation is that we used DSM-12D only in the second-wave survey. In the baseline survey, THI-D was used, and those who had depressive symptoms on this measure were excluded. The forth limitation is that the survey was conducted in limited area, and we did not collect information during the follow up period, thus the data were limited in the baseline and second-wave surveys. The last limitation is that there were no items addressing life events and economic problems in this survey. A previous study has shown that life events and economic problems are important factors in the development of depressive symptoms [51].

## Conclusions

We conducted a 7-year longitudinal survey to investigate whether health status and lifestyle factors present risks for the development of depression in community residents between the ages of 40 and 69 years. We found a gender difference in the risk factors predicting the development of depression. Chronic diseases and a lack of physical activity were the risk factors for men; poor perceived health, a BMI of 25 or greater, sleeping more than 9 hours and smoking were the risk factors for women. Preventive measures for depression must therefore take gender into account.

## Competing interests

The authors declare that they have no competing interests.

## Authors' contributions

HT was involved in data analysis and interpretation of the results, in addition to writing the manuscript. YS and SS established the concept and design of the Komo-Ise cohort study and carried out the data collection. MN contributed statistical analysis and interpretation of the results. HK supervised the data analysis and contributed to interpretation of the results and editing the manuscript. All authors contributed the interpretation and discussion of the results. They read and approved the final manuscript. The authors have no potential conflicts of interest to be disclosed.

## Pre-publication history

The pre-publication history for this paper can be accessed here:

http://www.biomedcentral.com/1471-244X/11/20/prepub
